# Chronic Intermittent Abdominal Bloating and Change in Bowel Habit: An Eight Year Diagnostic Problem Associated with Intra-Abdominal Adhesions

**DOI:** 10.7759/cureus.294

**Published:** 2015-08-03

**Authors:** Katherine Williams

**Affiliations:** 1 Section of Surgery, Imperial College London

**Keywords:** bloating, laparoscopy, endoscopy, intermittent bowel obstruction, adhesions

## Abstract

Abdominal bloating is a common clinical presentation and can impact significantly on the quality of life. It can be functional or signify more serious pathology.

We present a case presentation of a 38-year-old man who presented with recurrent episodes of abdominal bloating and severe debilitation for many years. He had suffered weight loss and was unable to tolerate solids. Ten years ago, he underwent an emergency laparotomy for sigmoid volvulus. In the presence of anaemia, normal imaging, and normal endoscopy, a laparoscopy was performed. At surgery, several adhesional bands were identified and resected. He had an uncomplicated recovery.

Acute and chronic adhesional bowel obstruction are common presentations and carry a significant morbidity and mortality. Elective laparoscopy can be a valuable tool for diagnostic and treatment purposes. Pathology should be suspected in cases where weight loss is a feature.

## Introduction

Abdominal bloating is a common patient presentation, estimated to affect ~30% of the general population [1-3] and can impact significantly on the quality of life [4]. It can be functional or signify more serious pathology. 

## Case presentation

A 38-year-old gentleman presented to the surgical clinic as a physician referral. He had been suffering for eight years with frequent episodes of abdominal bloating, nausea, weight loss, and constipation. Every time he consumed solid food his abdomen would distend to such an extent he would have to retire to bed until the episode settled. He would then pass a large amount of gas and his abdomen would deflate. He had been on a fluid diet for the last twelve months but had not been admitted to hospital during this time. His bowel habit was irregular and had not noticed blood in his stool. Of note, he had undergone an emergency laparotomy ten years ago for sigmoid volvulus whilst on holiday abroad, although he was not aware of exactly what operation had been performed, and no paperwork pertaining this was available. He was otherwise fit and well and took no regular medications. He had no family history of cancer or inflammatory bowel disease. On examination, he was cachectic with a well-healed midline laparotomy scar. He had no hernias and rectal examination was normal.

### Investigations

Full blood count revealed a normocytic anaemia (Hb10.9), and urea, electrolytes, and thyroid function were normal. Inflammatory markers were not raised. An abdominal x-ray showed prominent small bowel loops only. Upper and lower gastrointestinal endoscopy were reported as normal, as was an ultrasound and computerised tomography of his abdomen and pelvis.

### Differential diagnosis

- Functional bowel disorder

- Intermittent mechanical obstruction

### Treatment

**Figure 1:**
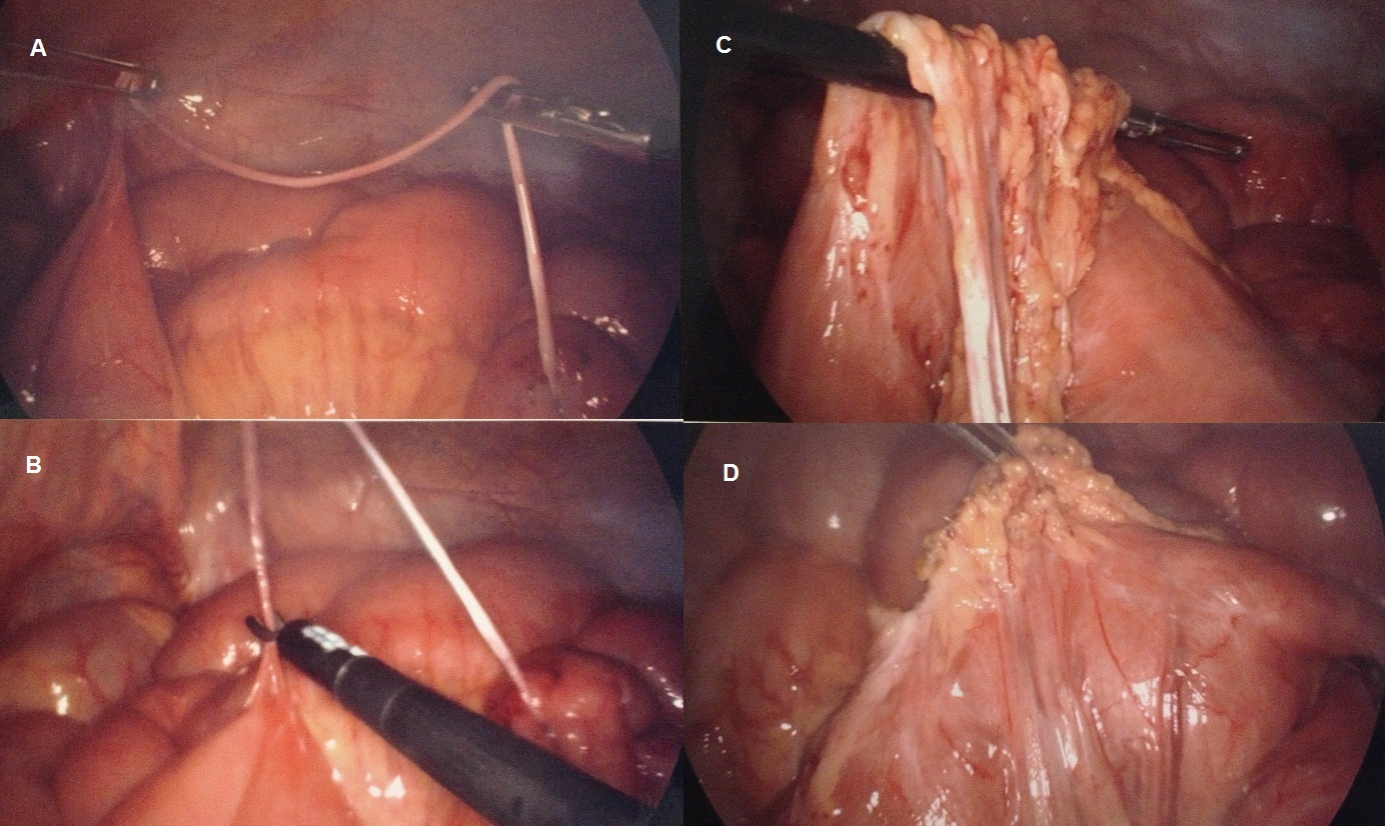
Laparoscopy findings (A) Thin adhesional band between colon and small bowel; (B) Band excision with laparoscopic scissors; (C) Dense adhesional band involving multiple regions of bowel; (D) Band excision and adhesiolysis

Informed patient consent was obtained for his treatment. This gentleman elected to undergo a laparoscopy during which two adhesional bands were found and resected. Multiple loops of small and large bowel were freed from adhesions to the abdominal wall and to each other (Figure 1). All bowel was viable and healthy. Sigmoidopexy sutures were noted.

### Outcome and follow-up

The patient was discharged home within 12 hours. He successfully ate a bowl of breakfast cereal before leaving the ward, a feat he had previously been unable to perform. He continued to increase his diet at home, and at six-month follow-up had put on weight.

## Discussion

The incidence of intra-abdominal adhesions following surgery is estimated at 67-100% [5-6]. They comprise of fibrous scar tissue, usually following surgical trauma or infection, and as yet, preventative measures have failed to develop [7]. Adhesions are associated with small bowel obstruction, chronic pain, and infertility [8]. Elective open or laparoscopic adhesiolysis can improve symptomatology, but can cause more adhesions to form. Acute episodes of small bowel obstruction thought to be due to adhesions, in the absence of an irreducible hernia, peritonism, or clinical shock, may be treated conservatively with nasogastric decompression and intravenous fluids. Surgical intervention, in the form of laparotomy or laparoscopy, is advised if patients do not respond to conservative treatment. Emergency surgery may be required if an ischaemic bowel is suspected. The mortality associated with acute adhesional bowel obstruction has been estimated at ~10% [9].

Abdominal distension and change in bowel habit is a common presentation to both physicians and surgeons and, in some cases, can be extremely debilitating for patients. This patient had been suffering from an undiagnosed bowel complaint for eight years. Diagnosis and management can be challenging, and all cases should be approached in a logical fashion. Intolerance of solids and weight loss should alert one to an organic pathology, and should be investigated as a matter of urgency. If symptoms suggesting colorectal cancer are present (see Table 1), recently published UK guidelines recommend urgent referral to be seen within two weeks (the “two-week rule”). If previous abdominal surgery has been performed, the presence of adhesions should be strongly suspected, and a laparoscopy in selected cases may be advised [10]. A functional bowel disorder should only be diagnosed if other pathology has been reasonably ruled out, and the Rome III criteria have been fulfilled [11].

**Table 1:** Symptoms prompting urgent referral for suspected colorectal cancer

Aged ≥ 40 years	Unexplained weight loss and abdominal pain
Aged ≥50 years	Unexplained rectal bleeding
Aged ≥60 years	Iron deficiency anaemia or change in bowel habit or presence of faecal occult blood

## Conclusions

Recurrent abdominal bloating is common, and a careful history and examination is paramount. Pathological causes should always be suspected if the diagnosis is uncertain. Adhesional obstruction after laparotomy is frequent, and clinical management depends on the patient presentation. It can be treated conservatively or surgically. Intermittent or subacute small bowel obstruction may not present in a classical way, and a high index of suspicion will aid in diagnosis.
